# A nomogram model for determining optimal patients for local therapy in metastatic prostate cancer: a SEER database-based study

**DOI:** 10.1186/s12894-023-01177-x

**Published:** 2023-01-31

**Authors:** Lin Yang, Sheng Li, Xiaoqiang Liu, Jiahao Liu, Fuchun Zheng, Wen Deng, Weipeng Liu, Bin Fu, Jing Xiong

**Affiliations:** Department of Urology, First Affiliated Hospital of Nanchang University, Nanchang, 330000 China

**Keywords:** Nomogram, SEER, Metastatic prostate cancer, Radical prostatectomy, Radiotherapy

## Abstract

**Background:**

Numerous studies have shown that local therapy can improve long-term survival in patients with metastatic prostate cancer. However, it is unclear which patients are the potential beneficiaries.

**Methods:**

We obtained information on prostate cancer patients from the Surveillance, Epidemiology, and End Results database and divided eligible patients into the local treatment group and non-local treatment group. Propensity score matching (PSM) was used to reduce the influence of confounding factors. In the matched local treatment (LT) group, if the median overall survival time (OS) was longer than the Nonlocal treatment (NLT) group, it was defined as a benefit group, otherwise, it was a non-benefit group. Then, univariate and multivariate logistic regression were used to screen out predictors associated with benefits, and a nomogram model was constructed based on these factors. The accuracy and clinical value of the models were assessed through calibration plots and decision curve analysis.

**Results:**

The study enrolled 7255 eligible patients, and after PSM, each component included 1923 patients. After matching, the median OS was still higher in the LT group than in the NLT group [42 (95% confidence interval: 39–45) months vs 40 (95% confidence interval: 38–42) months, *p* = 0.03]. The independent predictors associated with benefit were age, PSA, Gleason score, T stage, N stage, and M stage. The nomogram model has high accuracy and clinical application value in both the training set (C-index = 0.725) and the validation set (C-index = 0.664).

**Conclusions:**

The nomogram model we constructed can help clinicians identify patients with potential benefits from LT and formulate a reasonable treatment plan.

## Background

Prostate cancer(Pca) is the second most commonly diagnosed cancer and the sixth leading cause of cancer death among men worldwide, with an estimated 1,276,000 new cancer cases and 359,000 deaths in 2018 [1]. Distant metastasis is one of the main reasons for the high mortality rate of prostate cancer because metastatic prostate cancer(mPca) is currently considered incurable [2]. Androgen deprivation therapy (ADT) has remained the standard of care for patients with the metastatic disease since 1941 when Charles Huggins demonstrated that endocrine therapy could improve survival in patients with mPca [3]. Despite the rapid development of ADT drugs in recent years, tumor heterogeneity and acquired drug resistance are still important factors affecting the long-term survival of mPca patients [4].

In the treatment of malignant tumors, local treatment of the primary tumor is also an important treatment modality, which can even improve the survival rate of patients with distant metastatic disease [5]. Then, for mPca patients, local treatment (LT: radical prostatectomy or radiotherapy) of the primary tumor may bring survival benefits to selected patients, as the study by Stephen et al. has demonstrated [6]. At the same time, another key question is which patients with mPca can benefit from LT to improve overall survival. Although the study by Nicola Fossati et al. explored this question [7], we still lack a simple and useful model to screen patients with mPca who can benefit from LT.

Therefore, to aid clinical decision-making, we intend to utilize the SEER database to construct a nomogram model to screen the best mPca patients for LT.

## Method

### Patients

Patient information was acquired by the National Cancer Institute SEER database, which was accessed via the SEER*Stat software (Version 8.4.0; Username: 13084-Nov2021). Ethics committee approval is not required for this study as we have been granted access to the SEER database.

In our study, we screened 632,035 patients according to the year of diagnosis (2004–2015), and primary tumor site (Primary Site—labeled: C61.9), and then formulated inclusion and exclusion criteria according to the study requirements. Inclusion criteria: (1) Patients with distant metastases. (2) Received radical prostatectomy (code: 50, 70, 80) or radiotherapy. Exclusion criteria: (1) TNM stage was unknown. (2) The Gleason score was unknown. (3) Prostate-specific antigen (PSA) was unknown. (4) Cancer-specific death was unknown. (5) Undergoing partial resection or destruction of the prostate gland (code: 10–30). Ultimately, our study enrolled 7255 eligible patients.

### Variable definitions

We divided patients into different groups based on baseline characteristics and clinicopathological information: age (≤ 65 years vs > 65 years), race (White vs others), PSA (ng/ml: PSA ≤ 20 vs 20 < PSA ≤ 50 vs 50 < PSA ≤ 80 vs 80 < PSA), Gleason score (< 7 vs = 7 vs > 7), T stage (T1 vs T2 vs T3 vs T4), N stage (N0 vs N1), M stage (M1NOS vs M1a vs M1b vs M1c). LT was defined as receiving radical prostatectomy or radiotherapy, and NLT was defined as not receiving radical prostatectomy and radiotherapy.

### Statistical analysis

We divided patients into the LT group and the NLT group according to whether they received local therapy or not. Categorical variables were expressed as frequency (percentage), and compared using the Chi-square test. To minimize the influence of confounding factors on selection bias, propensity score matching (PSM) was performed. Covariates were included (age, race, PSA, Gleason score, TNM staging) for a 1:1 match with a caliper value of 0.05. The statistical software used was SPSS (26) and R software (4.1.3, http://www.R-project.org). Nomograms, calibration plots, and DCA were generated using the 'rms', 'foreign', 'survival', and 'rmda' packages, and bilateral test *p* < 0.05 was considered statistically significant.

### Construction and verification of nomogram model

The survival probability of the two patient groups was compared with the Kaplan–Meier curve. The LT group was then divided into a benefit group and a non-benefit group by comparing the median survival time of the two groups. The benefit group was defined as patients in the LT group whose median survival time was greater than that in the NLT group. Meanwhile, the matched LT group (n = 1923) was randomly divided into a training set (n = 1358) and a validation set (n = 565) in a ratio of 7:3.To identify factors associated with benefit, variables with *p* < 0.05, in univariate logistic regression, were included in multivariate logistic regression. Finally, nomograms were constructed using data from the training group based on variables screened by multivariate logistic regression.

For the construction of the predictive nomogram, we utilized the concordance index (c-index) and calibration curves to evaluate the application ability of the nomogram model in both the training and the validation sets.

### Clinical application

In addition, we compared the utility of the new nomogram model with traditional TNM staging in clinical decision-making by decision curve analysis (DCA). The nomogram was used to calculate the total score of each patient in the training set and validation set, and they were divided into two groups (high-benefit group vs low-benefit group) according to the score. The Kaplan–Meier curve was then used to compare the overall survival (OS) rates of the two groups to further validate the clinical efficacy of the model.

## Result

In our study, after screening for inclusion and exclusion criteria, a total of 7255 patients were recruited, including 1933 (26.6%) in the LT group and 5322 (73.4%) in the NLT group. After PSM, each group contained 1923 patients. The baseline characteristics of the two groups of patients before and after PSM are shown in Table 1. Before matching, there were significant differences in age, race, PSA, Gleason score, T stage, N stage, and M stage between the two groups. After matching, baseline characteristics between the two groups were balanced, reducing the effect of confounding factors.

**Table 1:** Clinicopathological characteristics of recruited patients

Variable	Before PSM		*p* value	After PSM		*p* value
	Nonlocal treatment (n = 5322,73.4%)	Local treatment (n = 1933,26.6%)		Nonlocal treatment (n = 1923,50%)	Local treatment (n = 1923,50%)	
Age(years)			< 0.001			0.012
≤ 65	1807 (34.0)	797 (41.2)		711 (37.0)	787 (40.9)	
> 65	3515 (66.0)	1136 (58.8)		1212 (63.0)	1136 (59.1)	
Race			0.091			0.027
White	4014 (75.4)	1495 (77.3)		1543 (80.2)	1487 (77.3)	
Others	1308 (24.6)	438 (22.7)		380 (19.8)	436 (22.7)	
PSA(ng/ml)			< 0.001			0.001
PSA ≤ 20	1182 (22.2)	678 (35.1)		573 (29.8)	668 (34.7)	
20 < PSA ≤ 50	992 (18.6)	320 (16.6)		399 (20.7)	320 (16.6)	
50 < PSA ≤ 80	502 (9.4)	156 (8.1)		167 (8.7)	156 (8.1)	
PSA > 80	2646 (49.7)	779 (40.3)		784 (40.8)	779 (40.5)	
Gleason			< 0.001			0.031
< 7	142 (2.7)	70 (3.6)		53 (2.8)	66 (3.4)	
7	799 (15.0)	358 (18.5)		300 (15.6)	352 (18.3)	
> 7	4381 (82.3)	1505 (77.9)		1570 (81.6)	1505 (78.3)	
T-stage			< 0.001			0.003
T1	2046 (38.4)	645 (33.4)		545 (28.3)	645 (33.5)	
T2	1921 (36.1)	689 (35.6)		766 (39.8)	687 (35.7)	
T3	745 (14.0)	354 (18.3)		345 (17.9)	351 (18.3)	
T4	610 (11.5)	245 (12.7)		267 (13.9)	240 (12.5)	
N-stage			0.896			0.732
N0	3576 (67.2)	1302 (67.4)		1283 (66.7)	1293 (67.2)	
N1	1746 (32.8)	631 (32.6)		640 (33.3)	630 (32.8)	
M-stage			0.211			0.017
M1NOS	154 (2.9)	54 (2.8)		28 (1.5)	54 (2.8)	
M1a	377 (7.1)	132 (6.8)		136 (7.1)	132 (6.9)	
M1b	4077 (76.6)	1450 (76.6)		1493 (77.6)	1446 (75.2)	
M1c	714 (13.4)	297 (15.4)		266 (13.8)	291 (15.1)	

Categorical variables as frequency (percentage). *PSM* Propensity Score Matching, *PSA* prostate specific antigen

**Fig. 1:**
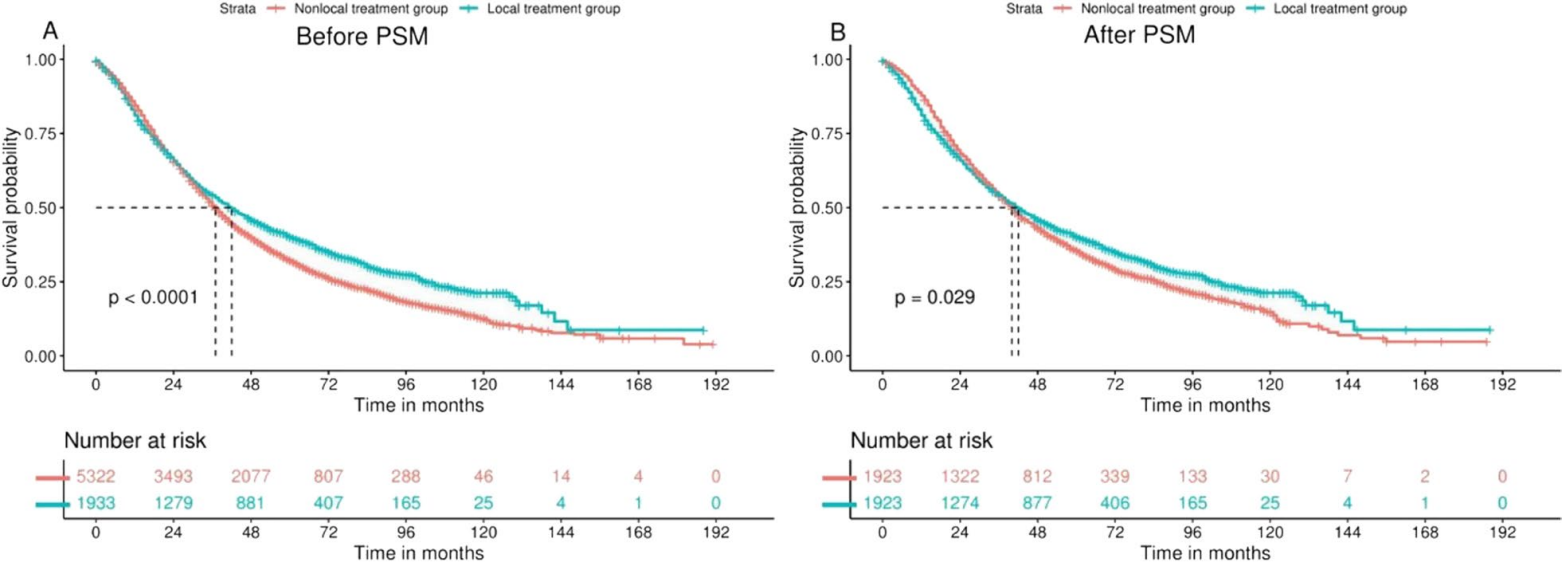
Kaplan–Meier plots show the overall survival of mPca patients in each group before and after PSM. **A** Before PSM, the prognosis of the LT group was better than that of the NLT group (42 months vs 37 months, *p* < 0.001); **B** After PSM, the prognosis of the LT group was still better than that of the NLT group (42 months vs 40 months, *p* < 0.001) = 0.03)

As shown in Fig. 1, we compared the OS of the LT and NLT groups using the Kaplan–Meier curve. Before PSM, the median OS of the LT group was significantly better than that of the NLT group [LT group: 42 (95% confidence interval: 39–45) months vs NLT group: 37 (95% confidence interval: 36–38) months, *p* < 0.001]. After matching, although the difference in median OS between the two groups narrowed [42 (95% confidence interval: 39–45) months vs 40 (95% confidence interval: 38–42) months, *p* = 0.03], it was still statistically significant.

To identify a potentially beneficial population of patients with metastatic prostate cancer receiving LT, we divided matched LT into a benefit group (median OS > 40 months) and a non-benefit group (median OS ≤ 40 months). Then, independent influencing factors affecting patients' benefits from LT were screened out by univariate and multivariate logistic regression analysis. As shown in Table 2, age, PSA, Gleason score, T stage, N stage, and M stage were independent predictors of patient benefit.

**Table 2:** Univariate and multivariate logistic regression analysis of local treatment groups after PSM

Variable	Univariable				Multivariable			
	OR	95%CI		*p* value	OR	95%CI		*p* value
Factors selected								
Age(years)								
> 65	1 [Reference]				1 [Reference]			
≤ 65	1.39	1.16	1.67	< 0.001	1.59	1.30	1.94	< 0.001
PSA(ng/ml)								
PSA > 80	1 [Reference]				1 [Reference]			
PSA ≤ 20	3.40	2.74	4.22	< 0.001	2.90	2.30	3.65	< 0.001
20 < PSA ≤ 50	2.10	1.61	2.74	< 0.001	1.83	1.39	2.41	< 0.001
50 < PSA ≤ 80	1.67	1.18	2.37	0.004	1.46	1.01	2.09	0.042
Gleason								
> 7	1 [Reference]				1 [Reference]			
< 7	3.44	1.96	6.03	< 0.001	2.63	1.45	4.75	0.001
7	2.46	1.93	3.14	< 0.001	2.05	1.58	2.66	< 0.001
T_stage								
T4	1 [Reference]				1 [Reference]			
T1	1.80	1.33	2.44	< 0.001	1.49	1.07	2.08	0.020
T2	1.49	1.10	2.01	0.010	1.26	0.91	1.75	0.166
T3	2.62	1.87	3.67	< 0.001	2.00	1.39	2.87	< 0.001
N_stage								
N1	1 [Reference]				1 [Reference]			
N0	1.30	1.07	1.57	0.008	1.32	1.05	1.65	0.018
M_stage								
M1c	1 [Reference]				1 [Reference]			
M1NOS	1.62	0.90	2.91	0.106	1.26	0.68	2.36	0.465
M1a	5.93	3.70	9.51	< 0.001	4.95	3.00	8.18	< 0.001
M1b	1.82	1.40	2.36	< 0.001	1.72	1.31	2.27	< 0.001
Factors not selected								
Race								
White	1 [Reference]							
others	0.94	0.76	1.16					

*PSM* Propensity Score Matching, *PSA* prostate specific antigen, *OR* odds ratio, *CI* confidence interval

**Fig. 2:**
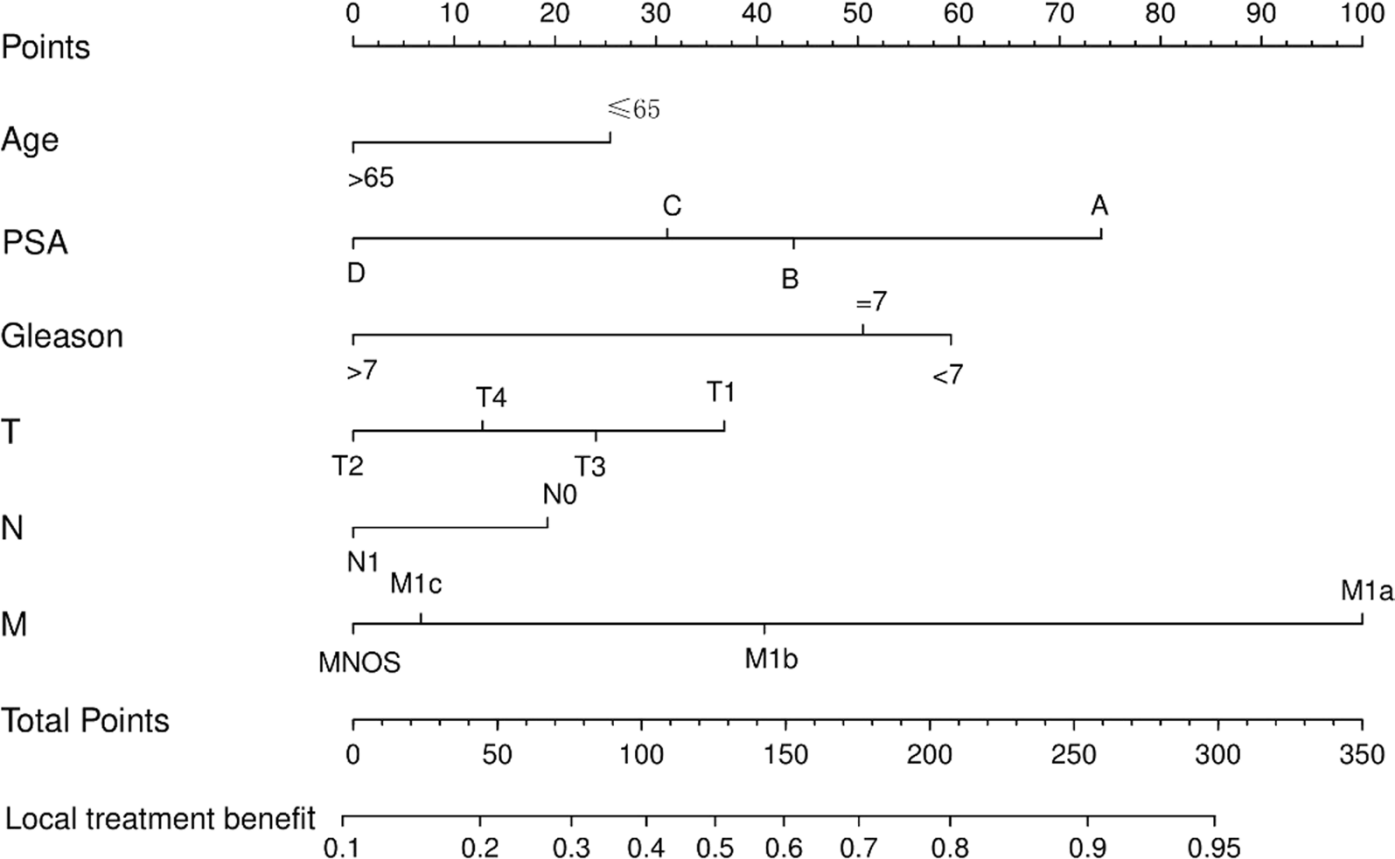
A nomogram model based on age, PSA, Gleason score, and TNM staging. The calculated total score corresponds to a probability greater than 0.5, indicating that the patient could benefit from LT

**Fig. 3:**
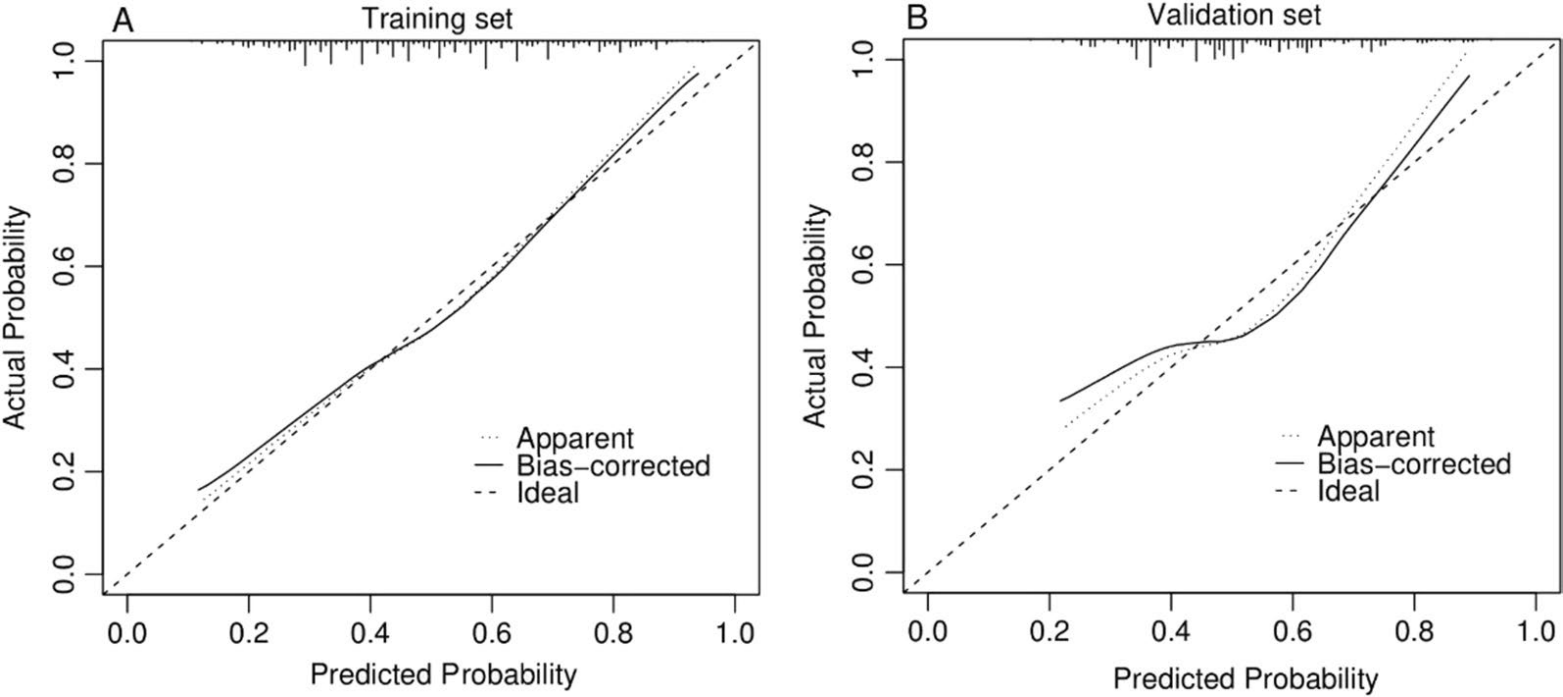
Calibration curves for the training set (**A**) and validation set (**B**). **A** C-index = 0.725; **B** C-index = 0.664

On this basis, we construct a Nomogram prediction model based on the training set (Fig. 2) and evaluate the accuracy of the model through the validation set. The model could well identify patients suitable for LT in the training set (C-index = 0.725) and validation set (C-index = 0.664). In addition, the calibration plots of the training and validation sets show that the model’s predicted probabilities are largely consistent with the actual probabilities (Fig. 3).

**Fig. 4:**
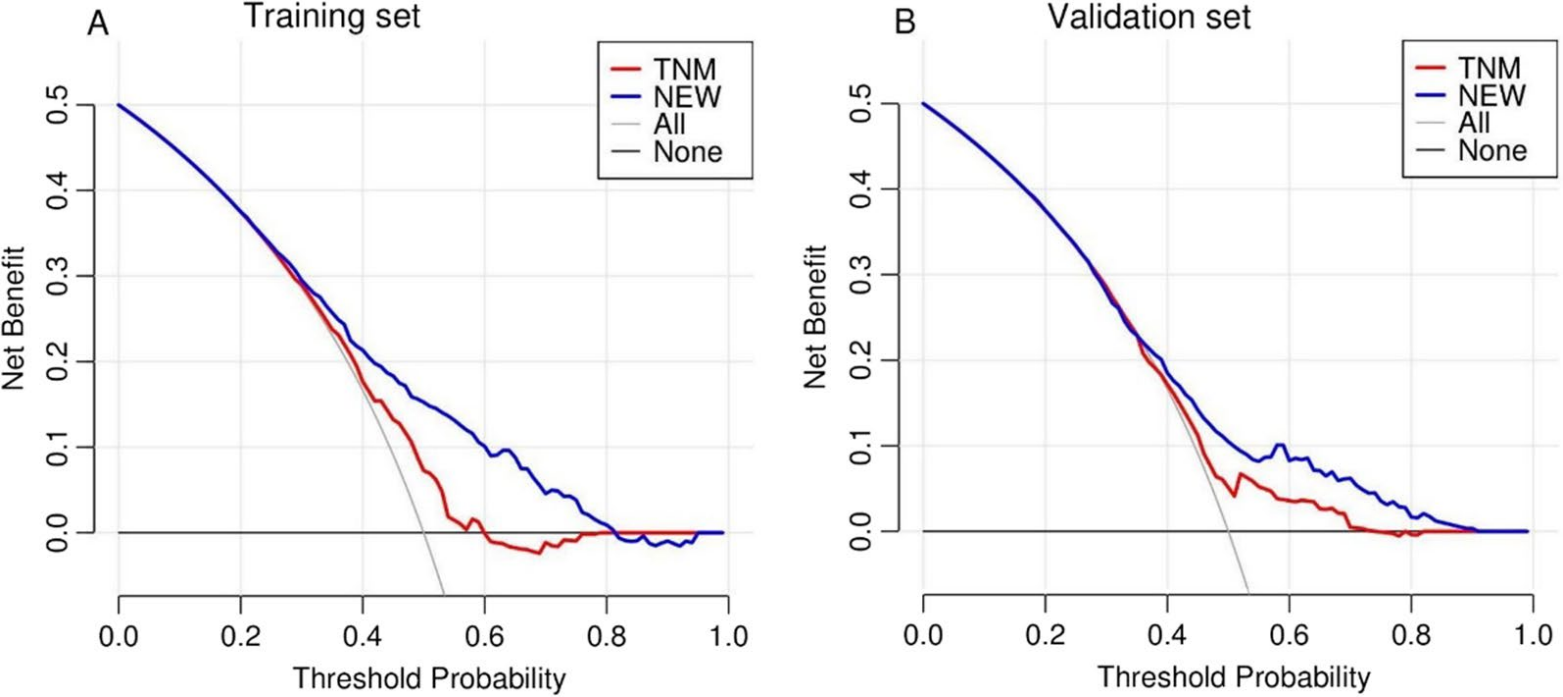
DCA curves of the training set (**A**) and validation set (**B**). The clinical value of the nomogram model is significantly better than the TNM staging

TNM staging is the most commonly used model in clinical practice. To verify that the new nomogram model has a higher clinical value, we plotted the DCA curves of the training set and validation set respectively (Fig. 4). Obviously, the area under the curve of the nomogram model is significantly larger than that of TNM staging, which can better identify the potential benefit population.

**Fig. 5:**
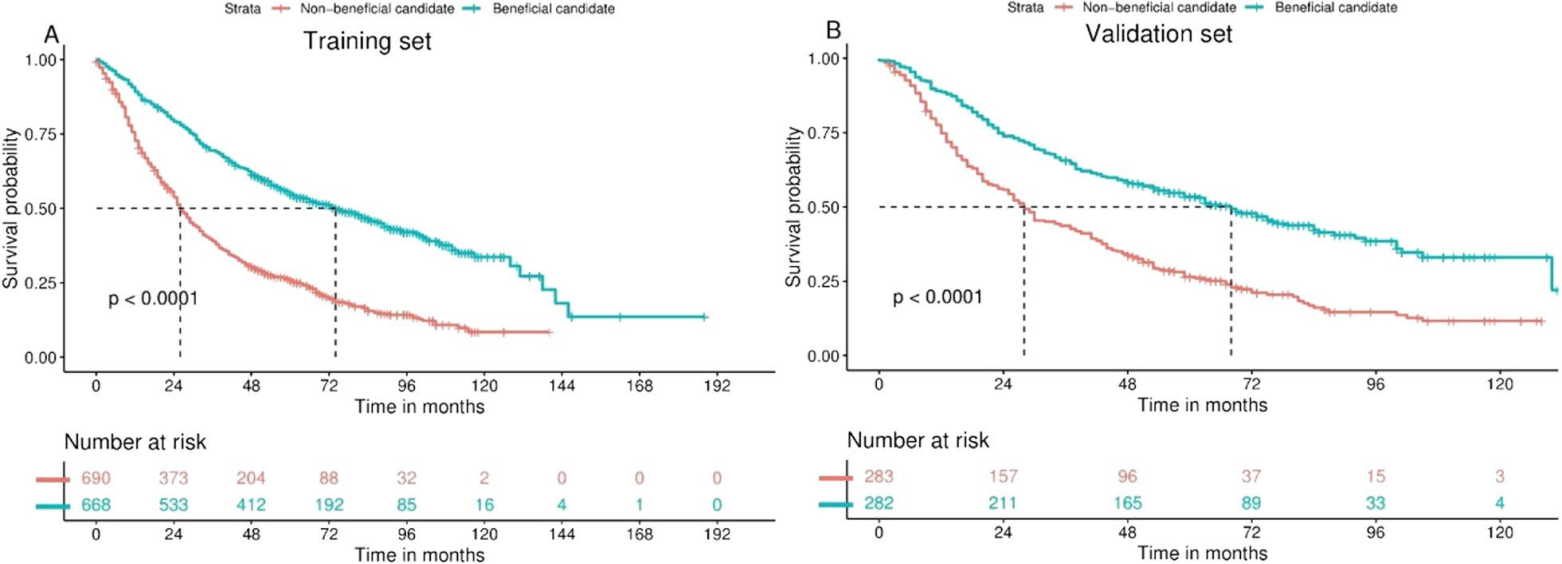
Kaplan–Meier plots of beneficial candidates and non-beneficial candidates in the training set (**A**) and validation set (**B**). The corresponding probabilities were calculated according to the nomogram. If *p* > 0.5, it is defined as a beneficial candidate, otherwise, it is a non-beneficial candidate

Moreover, to further validate the clinical value of the nomogram model, we defined a nomogram-predicted probability > 0.5 as a beneficial candidate and vice versa as a non-beneficial candidate. The predicted probability = 0.5 corresponds to a total score of approximately 125, splitting the training and validation sets into two groups (score > 125 vs score ≤ 125). The Kaplan–Meier curve was then used to compare the OS of the two groups (Fig. 5), and the results showed that the median OS of the beneficial candidate group was significantly higher than that of the non-beneficial candidate group (training set: 74 months vs 26 months, *p* < 0.001; validation set: 68 months vs 28 months, *p* < 0.001).

## Discussion

The treatment of prostate cancer, especially the treatment of patients with metastatic prostate cancer, is developing rapidly, and local treatment for the primary tumor site is a hot spot of concern. In our study, we found that radical prostatectomy and radiotherapy improved overall survival in selected patients with mPca. To accurately identify mPca patients who could benefit from LT, we developed the first SEER database-based nomogram model. Our model includes readily available measures of age, PSA, Gleason score, and TNM stage. At the same time, we compared the clinical value of the nomogram model and traditional TNM staging and found that the nomogram model can more accurately identify patients with potential benefits from LT.

Although localized prostate cancer has a high long-term survival rate, once metastases appear, it is difficult to cure because of the lack of treatment options that can produce durable responses at the genetic and cellular biological levels [8]. In other words, when mPca patients progress from metastatic hormone-sensitive prostate cancer (mHSPC) to metastatic castration-resistant prostate cancer (mCRPC), ADT-based therapy will lose its original efficacy [9–11]. In recent years, some progress has been made in improving the overall survival of mCRPC patients, such as abiraterone, enzalutamide, docetaxel, and other drugs that can significantly prolong the survival of patients [12].

To explore more diversified treatment modalities, Chad A Reichard et al. proposed that local treatment of mCRPC patients is safe and feasible, and can maintain the quality of life [13]. Whether local therapy can improve the survival time of mPca patients has always been a hot spot of concern. This question was explored as early as 2013 by Stephen H. Culp et al., who argued that LT could confer a survival benefit in patients with mPca [6]. Giorgio Gandaglia et al. found that radical prostatectomy was safe and effective in selected patients after a follow-up of 11 patients with oligometastatic prostate cancer who received local therapy [14]. At the same time, Michael Chaloupka's research shows that RP does not reduce the healthy quality of life of mPca patients [15]. In other words, local therapy can allow patients to maintain their original quality of life while controlling tumor progression. Two recent prospective studies have also validated this point, Sarah Buelens [16] and Chris C Parker et al. [17] suggest that LT can improve long-term survival in patients with mPca without reducing the quality of life.

Our study also confirms that specific mPca patients can benefit from LT. The key question, then, is which mPca patients are potential beneficiaries. To this end, we constructed the first nomogram model to address this question, in which PSA, Gleason score, and M stage were the main predictors. It is well known that PSA levels are positively correlated with the malignancy of Pca. As Hiroaki Iwamoto's study [18] showed, the percentage of T3-4 patients continued to increase as PSA levels increased. In our model, patients with PSA < 20 ng/ml had the highest probability of benefiting from LT, that is, with the increase of tumor malignancy, the effect of LT on tumor control would gradually decrease. Zijian Tian's study [19] also pointed out that in patients with PSA levels of 4–10 ng/mL, radiotherapy can significantly improve the overall survival of patients with mPca.

Gleason score is the most important parameter to describe prostate pathology, and it is also closely related to the prognosis of patients [20]. According to our findings, among mPca patients, the vast majority (5886, 81.1%) had a Gleason score > 7. It is well known that as the Gleason score increases, the risk of death in prostate cancer patients also increases [21]. Moreover, the study by David D Yang et al. also pointed out that patients with Gleason scores of 9–10 are less sensitive to ADT [22]. Our study also confirms this point, for mPca patients, even receiving LT does not bring survival benefits to patients with high scores.

In the nomogram prediction model, distant metastasis was the strongest predictor, and both patients with distant lymph node metastasis (M1a) and bone metastasis (M1b) could benefit from LT. A randomized clinical trial [23] showed that radiotherapy improved overall survival in M1a patients, low metastatic burden patients with no more than 3 bone metastases and no visceral metastases, which was highly consistent with the predictions of the nomogram model.

At present, many studies [17, 24, 25] have confirmed that radical prostatectomy or radiotherapy can improve the long-term survival of patients with metastatic prostate cancer. However, how to simply and efficiently determine the potential benefit population of LT remains an unsolved problem. The nomogram model we constructed has high accuracy and clinical application value and may guide clinical decision-making and future implementation of prospective studies.

Nevertheless, our study still has some limitations. First, this is a retrospective study based on the SEER public database, which contains an observational data set with a large amount of missing data and limited follow-up time [26]. We excluded cases with many missing data, which led to potentially less accurate results for survival analysis. Second, the SEER database lacks information on specific metastatic sites and the number of metastases, and they are important factors affecting the prognosis of mPca patients. In addition, mPca patients with low metastatic burden and few tumor metastases may benefit from LT to improve overall survival. Therefore, the limited inclusion of variables in this study might lead to inaccurate multivariate logistic regression analysis results.

## Conclusion

Our study constructed a nomogram prediction model based on age, PSA, Gleason score, T stage, N stage, and M stage to guide clinicians to screen out mPca patients who can benefit from LT, and formulating for these patients more reasonable treatment options.

## Data Availability

Our data are sourced from the SEER database and are available if necessary (https://seer.cancer.gov/data/.). Therefore, no ethics committee approval was required for this study.
